# Only two sex forms but multiple gender variants: How to explain?

**DOI:** 10.1080/19420889.2018.1427399

**Published:** 2018-01-31

**Authors:** Arnold De Loof

**Affiliations:** Functional Genomics and Proteomics Group, Department of Biology, KU Leuven-University of Leuven, Leuven, Belgium

**Keywords:** Calcigender, Calcium homeostasis, electrome, homosexuality, imprinting, nature versus nurture, reproduction, sexual behaviour, sex hormones

## Abstract

Are *sex* and *gende*r interchangeable terms? In classical biology, both are sometimes but not always used on an equal basis for some groups of animals. However, for our own species the *Homo sapiens,* they are not. A major question is why are there only two types of gametes (sperm- and egg cells), two types of sex steroids, (androgens and estrogens in vertebrates, and two types of ecdysteroids in insects), while the reproduction-related behaviour of the gamete producers displays a much greater variability than just two prominent forms, namely heterosexual males and heterosexual females? It indicates that in addition to a few sex-determining genes ( = the first pillar), other factors play a role. A second possible pillar is the still poorly understood cognitive memory system in which electrical phenomena and its association with the plasma membrane membrane-cytoskeletal complex of cells play a major role (learning, imitation and imprinting). This paper advances a third pillar, that hitherto has been almost completely ignored, namely the cellular Ca^2+^-homeostasis system, more specifically its sex-specific differences. Differential male-female genetics- and hormone-based Ca^2+^-homeostasis with effects on gender-related processes has been named *Calcigender* before. It will be argued that it follows from the principles of Ca^2+^- physiology and homeostasis that all individuals of a sexually reproducing animal population have a personalized gender behaviour. Thus, subdividing gender-behaviours in hetero-, homo-, bi-, trans- etc. which all result from a differential use of the very same basic physiological principles, is too primitive a system that may yield false sociological interpretations.

## 1. Introduction

The existence of sex-related differences in Ca^2+^-homeostasis is well documented in some species. Some obvious examples of animals in which females extrude much more calcium from their body related to reproduction than males are birds (eggs with a calcareous shell) and mammals that produce milk, with its extraordinarily huge Ca^2+^-concentration of about 50 millimolar (mM) compared to the very low Ca^2+^-concentration of about 100 nanomolar (nM) in the cytoplasm of resting cells ([Ca^2+^]i. This represents a concentration gradient of 50,000 times more Ca^2+^ in milk or 20,000x in blood where the Ca^2+^ concentration amounts to about 2 millimolar. These vertebrates are not exceptions: also in other vertebrates and in invertebrates the amount of Ca^2+^ extruded through egg laying is always higher than through the ejaculation of sperm. Thus, at least during the reproduction process, differential sex-related Ca^2+^-homeostasis is the general rule. Steroid sex hormones play an important role, but the mechanisms involved are not yet fully understood [1]. Other hormones may also be involved. In vertebrates the main difference in sex steroids between males and females does not reside in the type of steroids but in their relative amounts. In vertebrates, the rule is that both males and females produce androgens (testosterone, dihydrotestosterone) and estrogens (in particular estradiol) but in different amounts. Females convert more testosterone into estradiol than males in which the aromatase enzyme system that governs this conversion is less efficient. As a result, males have higher androgen concentrations in their body and tissues than females do. The opposite is true for estrogens: higher in females. This classical endocrinology has been well documented for a long time. However, how the genetic- and endocrine male-female differences are causally related to behavioural-gender differences is only partially understood.

## 2. A reminder: Some introductory definitions

**Sexual reproduction** is reproduction involving the use of specialized sex cells, called gametes. Prokaryotes, e.g. bacteria, cannot form such cells. Eukaryotes can. There are only two types of gametes, sperm cells (spermatozoa) and egg cells. There are no intermediate types of sex cells between sperm- and egg cells. By convention the larger type (egg cells) is called the female gamete, and the individual that produces them is the **female**. The smaller sperm cells are called male gametes, and the producers are called **males**. These definitions of sexual forms, which date from before the term *gender* was introduced, do not include any reference to reproduction-related behaviour. Thus with respect to biological sex, one is either male or female. Individuals that have the two types of gonads, either occurring together or alternating, are called hermaphrodites.

**Heterosexual**: a male and a female feel attracted to members and the opposite (second) sex/gender form, and usually cooperate to produce an offspring involving the fusion of a sperm- and an egg cell.

**Homosexual**: individuals of the same sex form are more attracted to each other than to members of the other sex. Homosexuality occurs in many animal species.

**Gender**: as will be outlined later, Gender can have different meanings. Males and females behave differently, and furthermore, not all genetically male or female organisms display the typical reproduction-oriented behaviour typical for their genetic sex. Sex and gender are invariably properties of the whole body, meaning that all cells participate.

**Figure 1.**
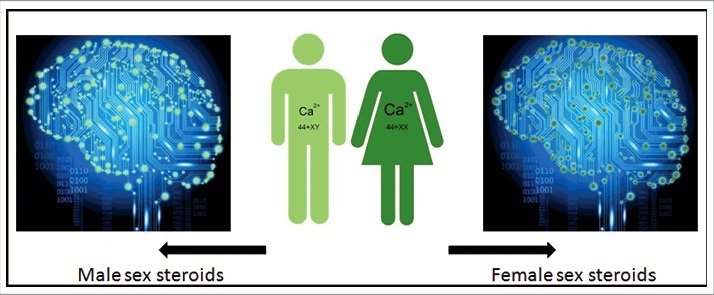
Cartoon illustrating the *Calcigender* paradigm as formulated by De Loof [2]. It says that it is a general principle in the Animal Kingdom that the Ca^2+^-homeostasis system displays substantial differences between males and females. Females extrude more Ca^2+^ than males do, a process in which sex steroids play an important role. Not only an 44+XX and 44+XY chromosomal configuration like in humans, but a variety of other sex-determining systems in other animals all yield such sex-related differential Ca^2+^-homeostasis, invariably with females extruding more Ca^2+^ through their reproduction-related activities than males. Because they are lipophilic, steroid sex hormones enter the membrane system of all cell types. They have effects on the Ca^2+^ system of all cells and tissues, the brain inclusive. The differences in green colour are only meant to indicate that there are differences in the complex whole Ca^2+^-homeostasis system, not to represent quantitative differences only. Indeed [Ca^2+^]i is very variable, e.g. due to short-lived Ca^2+^-puffs [3].

**Calcigender**: Males and females differ in their Ca^2+^ physiology (Fig. 1). Females remove more Ca^2+^ through their reproductive system than males. The term was introduced by De Loof [2].

## 3. Often overlooked in biology: Evolution has no goal whatsoever, things happen from physiologic necessity

One has to be aware that the widespread idea in many human populations and individuals that reproduction-related activities only have a meaning, or even that they are only permissible with the goal of procreation is a *sociological construct* that has its merits, but that does not follow from basic biological principles. The logical, not to say self-evident answer given to: Why do many, in particular higher organisms produce sperm and eggs? usually is: That is the condition to have (chances for) a progeny and for being evolutionarily successful. Yet, this answer implies that testes and ovaries *are aware* of the reproduction-oriented drive the whole organism might have. This cannot possibly be the case: ovaries and testes do not plan for the future by producing gametes. According to the current status of evolutionary theory [4,5], *there is no goal whatsoever in evolution*. Special sex cells did not come into existence with a specific goal. Gonads produce such sex cells and expel them from the body from physiologic necessity. But what is this necessity? The true reason is: To get rid of the abnormal-cancerous cells which the sex cells indeed are. If the cells can fuse after expulsion and give rise to a new individual of the next generation, OK, but that is not more than a fortuitous coincidence with an evolutionarily good outcome. The counterintuitive idea that sex cells are in some aspects abnormal requires some insight into physiologic archaeology in order to understand the mechanisms of sexual reproduction. Instead of planning for the future, which is inherent to a *goal*, one has to be aware that a positive outcome in the future in fact results from (random) mutations that bring some benefits for the progeny of the mutation-carrying individuals, with a later spreading of resulting beneficial traits into the population.

## 4. When and how did sexual reproduction come into existence? An intriguing, counterintuitive story

This type of reproduction only occurs in eukaryotes, thus in animals, plants and Fungi, not in prokaryotes (Archaea, bacteria). It is a relatively late event in the course of evolution as it took at least one billion (or more) years after the coming into existence of the very first prokaryotic cells. The symbiotic theory of Lynn Margulis [6] best explains how this might have happened. According to De Loof et al [7]., sexual reproduction, in its very evolutionary origin, likely results from an *ancient bacterial infection* of an early eukaryotic cell ( = a cell with a nucleus) by a prokaryotic organism, probably the premitochondrion, that managed to enter the proto-eukaryotic cell and to become a functional permanent resident. It evolved into the mitochondrion present in all contemporary eukaryotic cells. As is well documented in the fruit fly *Drosophila melanogaster* (references in [7]) a secretion product of the mitochondria, namely large ribosomal (mtlr) RNA, the Nanos protein, along with other factors, meant that some cells of a very young developing embryo could no longer make contact with other cells [8,9]. One of the effects is that the cells of the germline become immunologically alien to the normal somatic cells, and thus are experienced as intruders that have to be combated. Throughout life, these cells and their progeny remain isolated in the body as the *germ cell line*. Sexual reproduction can best be understood as a strategy of the innate immune system that prevents the body can prematurely die from the uncontrolled growth of these isolated egg cells with their giant dimensions, or from the production of very large amounts of sperm cells. Thus in this view, sexual reproduction results from an evolutionary ancient bacterial-type infection, with sub-lethal effects. Thus it is a mild disease [7].

## 5. The genetics underlying Sex

To date, a variety of genetic mechanisms are known that in different groups of eukaryotes are causal to the appearance of males and females [10,11]. That gives the impression that sexual reproduction emerged more than once in evolution without making use of a pre-existing universal genetic signalling pathway. That such a very complex physiological process could independently arise twice or even more times but always yielding sperm- and egg cells with a very similar physiology is highly improbable. In my opinion, we are still missing the mechanism that links the different strategies that became operational in the course of evolution. For example, in mice and most other mammals one gene directs sexual differentiation into males and females in mammals, namely the SRY gene [12,13]. The system is more complicated in females [14]. The SRY gene is located on the Y-chromosome and upon activation, it directs the individual into the male direction. However, Monotremes which are also mammals but which still lay eggs, do not have the SRY gene but nevertheless differentiate into males and females. In insects several mechanisms are known that direct sexual differentiation and gonad functioning [10,11,15]. In crustaceans, both an androgenic hormone (which is insulin-like [16]) and a peptidic female sex hormone (no similarity with other hormones in other species [17]) occur, this list not being exhaustive. Whatever the genetic variability in sex determination may be, and whatever endocrine mechanisms are at work, the final outcome in all sexually reproducing species is that females invariably extrude with their eggs much more Ca^2+^ than males do with their sperm: the Calcigender paradigm [2]). Apparently this outcome can be realized through diverse signalling pathways.

The fact that man and woman differ in so many traits may make us think that they have a high number of sex-specific genes. That is not necessarily so. The mechanism used in development is to keep the genome in the differentiated cells of an individual constant but to change, again and again the expression of some sets of genes in a cell-type/tissue specific way [18]. The same holds true for male-female differentiation: not many genes change, but their differential expression does. How powerful the cellular strategy can be is clearly illustrated by work of Uhlenhaut et al [19]. They discovered that cells predetermined to the formation of the ovary could be induced to transdifferentiate into a testis by an inducible deletion of only one gene, namely the forkhead transcriptional regulator FOXL2. Such deletion in adult ovarian follicles leads to immediate upregulation of testis-specific genes including the critical SRY target gene Sox9. This way the production of androgenic sex steroids can also be induced.

## 6. Fertilization: The need for an often complex behavioural input by the gamete producers. Making heterogametes meet

Gametes are formed by a special type of cell division, called meiosis, which only occurs in the gonads, not in any other tissue. It only yields haploid cells, thus cells with only half the number of chromosomes of normal somatic cells. Such haploid cells cannot divide any more. Once gametes have been formed and are expulsed from the body, they can only genetically survive by fusing, usually but not always within a short time after their production, with another type of different size ( = the act of heterogametic fertilization). Thereby the normal diploid situation is re-established, and subsequent regular cellular multiplication by mitosis becomes possible. Fertilization is only possible if sperm and egg cells *are brought into each other's very close vicinity*. The multitude of ways to achieve this, in particular in free-living organisms, is, in my opinion, *the evolutionarily ancient origin and essence of gender*. Behavioural adaptations directing the way gametes have to follow to ensure fertilization becomes part of the reproduction scene. This raises the question: Which mechanisms govern this type of behaviour? Depending upon the prevailing environmental conditions, the *making heterogametes meet* can happen passively (e.g. in aquatic organisms like most fishes, corals etc., pollination with the aid of pollen carriers like the wind or insects etc. in plants), meaning that gender hardly has a meaning in such moderately stressful conditions. However, in harsher, e.g. dry conditions, it may require a specific sexual partner-seeking behaviour by the gamete producers. Sex pheromones are a widely used tool to bring up such behaviour. The origin of dominance of heterogametic over homogametic fertilization (e.g. fusion of an oocyte with its sister polar cell, an existing but rare possibility), may be seen as an evolutionarily successful strategy to avoid inbreeding.

## 7. Gender

If sex and gender would have the very same meaning in all sexually reproducing species, there should be no need for two terms: Sex would suffice. Gender does indeed have no meaning in the few species which only produce one type of gamete, which is egg-like, thus in the few species in which no males occur. Such species have special means to maintain the diploid status of their somatic cells. Gender requires the presence of males and females. But why is there need for two terms? In non-human animal research, gender is commonly used to refer to the biological sex of the animals. Thus in classical biology, the nature of gender is not a hot topic, and hardly ever have efforts been undertaken to come up with a good definition. The opposite situation prevails in the humanities, in particular since the 1960-ties, when some sociologists and historians started raising questions about the reasons why males and females behave so differently, why specific tasks were typically attributed to females or males, and why man and woman were not always treated as equals, e.g. in receiving the same pay for the same work/job. An answer like e.g. God had a different set of tasks for man and woman in mind (see e.g. the story of creation in the Book Genesis of the Bible, or other stories in other cultures) when He created the species *Homo sapiens* as heterosexual as He had done before in other species; was rightly no longer accepted as a valid argument. Even to date, defining gender remains tricky.

There is no generally accepted definition of gender, because the concept itself is not static but dynamic [20]. According to Weed [21] the meaning of gender depends on who uses the word, in what context, and for what ends. A few examples of definitions as used in medicine or in the humanities, in particular in sociology are:

• Gender: the behavioural, cultural, or psychological traits typically associated with one sex (Merriam-Webster Medical dictionary)
• Gender: is a constitutive element of social relationships based upon perceived differences between the sexes and gender is a primary way of signifying relationships of power (historian Joan Wallach Scott [22]).
• Gender: is the range of characteristics pertaining to, and differentiating between masculinity and femininity. Depending on the context, these characteristics may include biological sex (i.e. the state of being male, female or an intersex variation which may complicate sex assignment), sex-based social structures (including gender roles and other social roles), or gender identity [23]).
• Since 2011, the FDA [24] started using *sex* as the biological classification and *gender* as a person's self-presentation as male or female, or how a person is responded to by social institutions based on the individual's gender presentation.
• To my knowledge, no specific definition of gender emerged from basic studies in animal physiology and development.

These definitions illustrate that a triplet of basic elements is taken into account, namely biological sex, psychological gender, and social gender role. Gender is wider than sex. To date gender is mainly used in a human sociological context, with a considerable input from feminist theory and with little reference to basic principles of fundamental biology [20,23,25]. I am primarily interested in the uncovering which principles from animal physiology and development are responsible for the difference between sex and gender, and for enabling variability in gender forms.

### 7.1. The problem of extending *gender* to non-human species

Animal physiologists do not see how the majority of terms used in the definitions of gender formulated in the humanities, e.g. personality, relationships of power, gender role etc., can be linked to physiological processes, a necessity if one aims to use gender for other animal species as well, as is already sometimes done. Hence an approach that is different from the sociological one is needed in order to explain aspects of gender using the methodologies used the exact sciences (biology, chemistry, neurosciences etc.). Gender, implying specific reproduction-related roles for the producers of gametes must have come into being because of a physiologic necessity. *The primordial necessity was to bring heterogametes in each other's close vicinity so that fusion-fertilization became possible*. An individual that sheds its eggs or sperm cells haphazardly somewhere in nature where there is no chance for fusing with a matching heterogamete, will not become reproductively successful. Only individuals that manage to shed their gametes into a suitable environment have a chance for survival through a progeny. In my opinion, the very origin of gender dates back to the time that in a heterogametic environment some gamete producers acquired the ability to shed their gametes only in an environment that at least offered the possibility for participation in fertilization. This insight implies that group-wise living is very advantageous over solitary living. Furthermore, competing for attracting a suitable sexual partner is also an advantageous strategy.

### 7.2. Social insects: Not *gender*, but behavioural groups or *castes*

**Figure 2.**
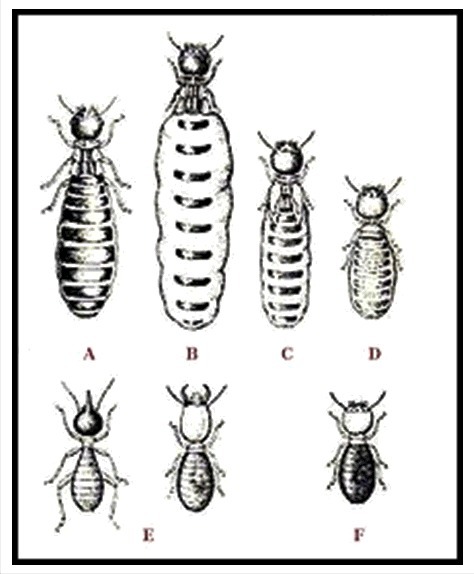
Caste system of termites: A – King; B – Queen; C – Secondary Queen; D – Tertiary Queen; D – Soldier; E – Worker. From [27].

People usually tend to consider insects, even the very strictly organized social insects with their caste systems, as primitive and simple creatures compared to themselves, the (supposedly superior) *Homo sapiens*. From an evolutionary point of view this is incorrect, and even the opposite may be true because of the unique combination of features present in insects. Arthropods and vertebrates descend from a common ancestor in which the basic principles of physiology and biochemistry were already very well developed, and which have been very well conserved in evolution. Insects and other arthropods have a short life cycle and often many descendants. Eusociality [25] is very well developed in some insect species. This means that in an evolutionary perspective, they are (multi)million generations ahead of us in refining their adaptation to their habitats. Insects are specialists in miniaturization (small body size) and in highly efficient physiological systems. In social insects, the term *castes* is used (Fig. 2) to denominate different polymorphisms, each with a given specific labour task [26,27]. In social insect issues the term *gender* is not used, although from an anthropomorphic sociological point of view, this term could be applied to some aspects of polymorphism, e.g. behaviour. Hormones, e.g. juvenile hormone with its link to Ca^2+^-homeostasis [3] play a major role in caste determination in termites. The hierarchical/caste organization in social insects is mainly Nature and much less Nurture [28].

## 8. The crucial role of behaviour in gender determination

### 8.1. The underlying basic principle

In my opinion the central question in biology for trying to unravel the mechanisms enabling gender might be: First: How do organisms, in their role as gamete-producers, behave, not only during the reproductive period but throughout life? Second: How are gamete-producers perceived by other members of the population/society? This question mainly deals with behavioural aspects. Behaviour, being the totality of movements an organism makes, rests upon multiple pillars: genetics ( = input through the DNA memory system)), learning/imitation [29] and motivation ( = input through the cognitive memory system), muscle physiology in which differential Ca^2+^ homeostasis, hormones, and self-generated electricity carried by inorganic ions (the cell's self-generated electrome concept [30,31] play roles. Such combination promotes distinguishing specific heterosexual-homosexual-other gender forms. If the reproduction-related behaviour would only be determined by the same set of genes that govern gonad formation and differentiation, there would probably only be heterosexuality and no homosexuality. But homosexuality, bisexuality, asexuality etc. do occur.

Thus, the observed variability in behaviour must be caused by a more complex (set of) mechanism(s) than just the few genes that govern gonad differentiation. For animal physiologists, relevant questions are: If the sex-determining genes alone do not allow for the observed variability in reproduction-related behaviour, which additional mechanisms play a role? What can we learn from the numerous studies on the physiological basis of homosexuality in order to better understand gender? And above all, does the Ca^2+^-homeostasis system allow differences in gender-linked behaviour?

### 8.2. The crucial role of Ca^2+^ in behaviour: Principles of Ca^2+^-homeostasis and its endless variability

This section is only intended for readers who are not familiar with the role of Ca^2+^ in muscle contraction-relaxation, thus in the physiological fundamentals of behaviour.

**Figure 3.**
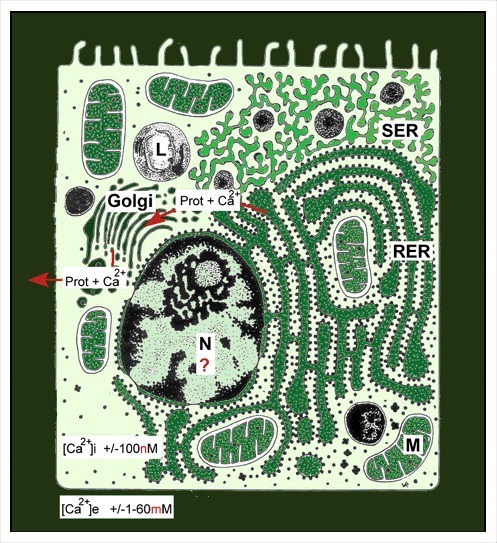
Schematic representation of the major Ca^2+^-gradients in cells. A Ca^2+^ concentration of 2 millimolar in blood is about 20,000 times higher than the 100 nanomolar Ca^2+^ concentration in the cytoplasm of unstimulated (resting cells). Intracellular Ca^2+^ gradients can also be built up inside cells. Ca^2+^-ATPases are enzymes that reside in membranes and which can actively transport Ca^2+^ ions against the Ca^2+^ gradient. This way, the Ca^2+^ concentration in the lumen of the endoplasmic reticulum can become much higher than in the cytoplasm. Sex steroids can influence this transmembrane gradient. If more Ca^2+^ enters the cell that can be extruded by Ca^2+^-ATPases present the plasma membrane, the rough endoplasmic reticulum is triggered to remove extra Ca^2+^ along with proteins [3]. Calcareous egg shells are one of the possible outcomes of such strategy. This figure is borrowed from [3].
• The Ca^2+^ homeostasis system is evolutionarily very ancient and displays a very high degree of variability. It is the most important signalling system in eukaryotic cells. It has been shaped to near perfection during a few billion years. The mode of action of numerous different signalling molecules (hormones, neurotransmitters etc.) involves some aspect(s) of Ca^2+^-homeostasis. The entry of Ca^2+^ into cells as well as the removal of excess Ca^2+^ from the cytoplasm is rigorously regulated. The key players are the different types of Ca^2+^-channels and of Ca^2+^-pumps, and ATP as energy source. *Because of this complexity, there cannot be two cells in a body with identical Ca^2+^-homeostasis systems*. For a brief introduction to the principles of Ca^2+^-homeostasis that includes some figures, see [2,3,32].
• Many hormones, sex steroids inclusive, use Ca^2+^ as their secondary messenger. In particular, many signalling molecules that bind to GPCRs (G-protein coupled receptors) do so. This means that upon binding of a ligand, a short-lived Ca^2+^ puff arises that contributes to signal transduction.
• There is also a role for the Ca^2+^-gradient on the activity of some enzymes present in the intracellular membranes. To understand the differential role of sex steroids, one should also keep in mind that some of the enzymes governing the biosynthesis of lipids and steroids (Fig. 3) reside in the membranes of the SER. Apparently, as long as muscle (and other cells) are not stimulated by neurotransmitters or hormones, the Ca^2+^- gradient inside-outside the lumen of the SER is maximal. In such conditions the cited enzymes are inhibited by the high Ca^2+^-concentrations. A lower gradient means that the inhibition of the enzymes needed for lipid or steroid synthesis are less efficiently inhibited, resulting in more lipid or steroid biosynthesis. In many mammalian species, males are bigger and more muscular than females, while females may more readily deposit lipids in some tissues which is beneficial for pregnancy and milk feeding. That is part of their gender phenotype. In insects, which use ecdysteroids as sex hormones, the opposite situation prevails, namely females are usually bigger. From what is known about the role of Ca^2+^ gradients in the SER, in particular in muscle cells, androgens seem to be more potent in keeping the Ca^2+^ gradient in the cytoplasm versus the concentration inside the lumen of the Ca^2+^ storing compartments (SER, RER) steeper in males, at least in mammals. The higher excretion of Ca^2+^ by females indicates that females, under the influence of their hormones, cannot maintain as steep trans-membrane Ca^2+^ gradients as males.
• Extrapolation to the nervous system as well? There is no reason to assume that differential male-female Ca^2+^ homeostasis that applies to the skin, muscle cells, mammary gland cells etc., would not apply to brain cells. Thus, it is logical to assume that the perception of the outside world and the ways to react to it can be different for males than for females. Steroid hormones certainly play a role, but not in an as clear-cut way that behavioural differences between males and females simply result from the sex-specificity of androgens and estrogens, as was thought decades ago [33-36]. Brain plasticity during development and other additional actors have to been taken into account [37-38]. Some are still missing. In my opinion, differential Ca^2+^ homeostasis is one of the actors in bringing about sex-gender specificity in reproductive behaviour and in imprinting [29]. Imprinting is essential in e.g. mother-baby recognition, and in making individuals aware of features that mark the opposite sex-gender [39]. Preference for the opposite-sex partners may arise in part through the organizing actions of sex steroids [40]. Depending upon the species, a variety of senses can contribute: smell, vision, touch etc. How imprinting is achieved at the subcellular level in brain cells, remains to be elucidated. As long as the basic principles underlying the storing and retrieving information in the cognitive memory system are not unravelled, imprinting will remain an enigma. Maybe imprinting results from a permanent fixation in the cognitive memory system of an electrical input from a very strong stimulation of some sense receptors aided by a Ca^2+^ puff. The plasma membrane-cytoskeletal complex with its special electricity-conducting properties of actin may play a key role [31].

• Ca^2+^ is an omnipresent ion that has both *very toxic and beneficial effects* in cells. Because the Ca^2+^ concentration in the external environment of cells is thousands of times higher than inside cells (Fig. 3), Ca^2+^ will leak into the cytoplasm through Ca^2+^ channels that are not completely closed, or that are forced to open occasionally under the influence of some neurotransmitters and hormones. Cells can tolerate low Ca^2+^ concentrations. Ca^2+^ can become toxic because at concentrations above 100 nanomolar (which is very low) it can change the spatial (3D) conformation of many macromolecules, in particular of some proteins. It is upon this principle that the sliding filament theory of muscle contraction is based. Muscle cells contain complexes of actin-myosin-troponin that can be in a relaxed- or in a contracted state. With respect to the Ca^2+^-concentration in muscle cells, the free Ca^2+^ concentration in the cytoplasm of relaxed muscle cells is very low (about 100 nanomolar (nM)). Muscle cells have an extended intracellular membrane system, the smooth endo/sarcoplasmic Reticulum (SER), in whose lumen high concentrations of Ca^2+^ can be temporarily stored. These concentrations can be as high as a few millimolar (mM). When under the influence of neurotransmitters a muscle cell is triggered, some Ca^2+^ passes the plasma membrane through Ca^2+^ channels. In addition, more Ca^2+^ is also very quickly released from the Ca^2+^ stored in the lumen of the SER. The resulting rise in cytoplasmic Ca^2+^ ( = a Ca^2+^ puff) makes that the proteins that make up the contractile system (actin and myosin slide along each other thereby causing shortening) contract.

## 9. Sex hormones: Remaining question marks in their mode of action

### 9.1. Important for physiology: Vertebrate-type sex steroids are lipophilic, thus moderately to not soluble in water

**Figure 4.**
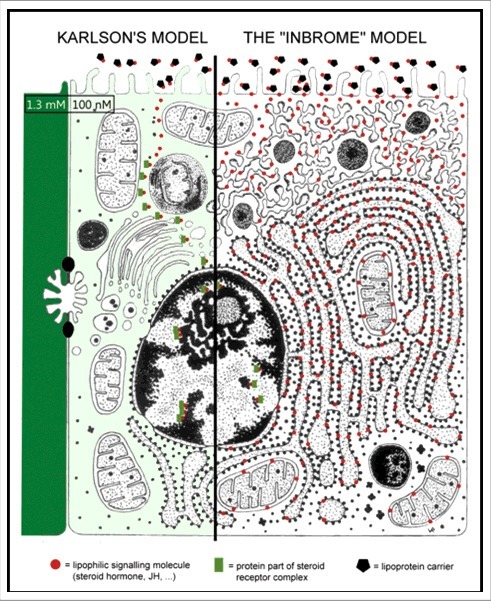
Schematic representation of the ways sex steroids and other lipophilic hormones may exert their actions. The classical hypothesis of Karlson and Sekeris [42] originally developed to explain the mode of action of the steroid moulting hormone of insects, says that the hormone passes the cell membrane unhindered, is transported somehow through the cytoplasm and finally ends up in the nucleus. There it controls the expression of selected genes. The moulting hormone occurs in two forms, ecdysone (more lipophilic) and 20-OH ecdysone (somewhat less lipophilic. Some ecdysteroids also function as sex steroids of insects [15]. The inbrome hypothesis says that lipophilic hormones, after have been delivered by a hormone-transporting blood protein, enter the plasma membrane, and next diffuse over the whole membrane system, internal membranes inclusive. If they meet membrane proteins with a matching binding site, e.g. ion channels in the plasma membrane, or ion pumps , e.g. Ca^2+^ -pumps in internal membranes, they may influence the ionic internal environment and the activity of some enzyme systems [3].

Gender-related behaviour is strongly influenced by the steroid hormones that are released from the testes and the ovaries, the so called sex steroids. This is very well documented in the literature. The hormones must have the right (stereo)chemical properties to be omnipresent in the body, thus in all cells. The fact that sex steroids are lipophilic/hydrophobic is a necessary property in this respect. Indeed, when lipophilic hormones that circulate in the blood after their release from their production site come in contact with the plasma membrane of cells, they will be transferred/jump from their carrier lipoprotein in the blood into the plasma membrane on the condition that the apolar properties of the membrane lipids better match those of the steroids than the lipid moiety of the carrier protein. Once the steroid has entered the membrane, it will start diffusing all over the whole system of all interconnected cellular membranes: the endoplasmic reticulum, Golgi, nuclear envelope etc. (Fig. 4). There it can come in contact with the different membrane-bound actors of the Ca^2+^-homeostasis system. In this respect, the well documented very fast system of Ca^2+^-induced Ca^2+^ release from internal storage sites of Ca^2+^ (the SER and mitochondria) is a very powerful tool to govern behavioural changes based on muscle contraction. Another important mechanism is the stimulation by Ca^2+^-influx of the release of neuropeptides that control steroidogenesis [41] and some aspects of behaviour. It remains to be further clarified how a steroid can exert a function when it is present in the plane of a membrane [3].

### 9.2. Insects and other arthropods use another type of sex steroids

In insects, the best studied invertebrate models in this respect, not vertebrate-type androgens and estrogens play a role as sex steroids, but ecdysteroids, the hormones that are best known for their role in moulting and in metamorphosis, but that concurrently act as sex hormones. Again two closely related molecular forms of steroids, ecdysone (E) and 20-hydroxyecdysone (20E), occur. According to [15] 20E is the functional equivalent of estrogens, and thus acts as the female sex steroid. In addition to its role as precursor of 20E, E is also thought to be a candidate androgenic steroid in insects [43], but experimental evidence is difficult to generate because upon injection, E is rapidly converted into 20E. That makes it difficult to distinguish between the effects of E and 20E.

### 9.3. Sex-specific differential titres of sex steroids

**Figure 5.**
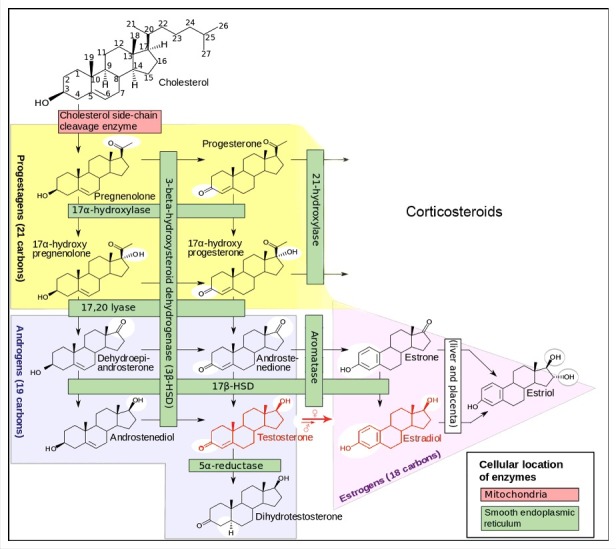
Biosynthetic pathway of steroid hormones in vertebrates. The relevant sex-steroids are in the squares with the red borders. Both males and females produce androgens and estrogens. The difference does no not reside in their types of sex steroids but in their titres in the blood. Females convert more testosterone into estradiol. Hence the testosterone titre is higher in males, while the opposite is true for estradiol. Modified after [45].

The chemical nature of estrogens and androgens has been known for almost a century [44]. Their biosynthetic pathway starting from cholesterol is also well documented (Fig. 5). The key point is that in vertebrates testosterone and estrogens are synthesized in both males and females, but their concentrations in the blood differ substantially. This is due to the greater conversion of testosterone into estradiol in females than in males. As a result the androgen titre is higher in males than in females. In other words, the male body is somewhat more *poisoned* (see later why this term is used) by testosterone than that of females. In females, the estradiol titre is higher: their body is more poisoned by estrogens. The physiological effects of both types of steroids are quite pronounced. However, if one looks at the molecular structure of testosterone and dihydrotestosterone and of estrone and estradiol, the differences look minimal. Estradiol and dihydrotestosterone are somewhat less apolar than their precursors (Fig. 5). Thus, to use an anthropomorphic vocabulary, testosterone will feel more comfortable in the lipid environment of the membrane than dihydrotestosterone for which the more watery nuclear environment may be more suitable. A similar reasoning also arises for estrone-estradiol. The more polar forms, estradiol and dihydrotestosterone which are commonly referred to as the most active forms of the sex steroids, apparently enter the nucleus (with the help of cytoplasmic steroid transporting proteins) more readily than their slightly more apolar precursors, and influence transcription (Fig. 4 left side) of selected genes.

### 9.4. Membrane receptors for steroid hormones: Hitherto a rather unsuccessful search

Steroid hormones not only control the expression of selected genes which is a slow process. Because the nucleoplasm is a watery environment, the steroids active here must be compatible with such environment. Some steroids (also) influence behaviour, thus the contractility of some muscles which is a much faster system. Here more apolar molecular forms are better suited to act *the inbrome way* (Fig. 4 right side). The speed of most muscle contractions so high that *de novo* protein synthesis which takes minutes at least, cannot be involved for the short term [46]. A faster, ionic-electrical system that operates at the membrane level is involved [30,31]. When muscle contraction-relaxation is involved, changes in intracellular Ca^2+^ gradients are at work. This points to the causal relationship between differential Ca^2+^-homeostasis and differential behaviour in a gender-type way. The well documented differences in Ca^2+^-homeostasis in some groups of animals, e.g. in birds with their calcareous egg shells, indicate that sex-specific hormones directly influence Ca^2+^ secretion. Females excrete more Ca^2+^ than males. Thus, sex steroids must affect at least some Ca^2+^-channels and pumps. Some *plasma membrane* receptors for estrogens, progesterone, and androgens are known. Receptor promiscuity has also been documented [47,48].

### 9.5. The SERCA-pump as a promiscuous receptor for lipophilic hormones (insect juvenile hormone, steroids…)?

The mode of action of transmembrane transport of Ca^2+^ by the SERCA pump is well documented. A key feature is *Ca^2+^-induced Ca^2+^-release*, which is a very powerful and very rapid signal-amplification system. It means that a small increase in [Ca^2+^]i, e.g. by activation of a GPCR that uses Ca^2+^ as secondary messenger, may cause the sudden release of Ca^2+^ from internal storage sites. The SERCA pump will quickly restore equilibrium. In their analysis of the mode of action of another type of lipophilic molecule, namely the farnesol ester juvenile hormone, De Loof et al [3,49,50]. pointed to the fact that the binding site of thapsigargin, a potent sesquiterpenoid blocker of the SERCA pump, might very well be the *intracellular* natural binding site of a variety of small lipophilic signalling molecules such as endogenous farnesol-derivatives as well as other lipophilic hormones. This may explain why the search for membrane receptors of both sex steroids and juvenile hormone has been relatively unsuccessful. Indeed, researchers did not look for these *intracellular membrane* proteins, or even if they did, they could not identify them with certainty. Nobody seems to have considered the possibility that an intracellular ion pump, in particular the SERCA-pump ( = sarcoplasmic reticulum Ca^2+^-ATPase) could act as a (probably promiscuous) receptor for lipophilic signalling molecules [49,50]. By controlling the activity of the SERCA, intramembrane signalling molecules may contribute to influence the steepness of the transmembrane Ca^2+^-gradients in the SER and RER, and by doing so, regulate the activity of a variety of enzymes which are embedded in these membranes.

### 9.5. Nuclear receptors for steroid hormones

This family of receptors is much better documented [48,51] than membrane receptors [47]. Some act as transcription factors and play a role in controlling which genes are switched on or off. For example, the synthesis of milk- and yolk proteins is controlled – in part – by female sex steroids. Androgens control the synthesis of some proteins in the male genital system and its accessory glands. Some also have anabolic effects, and can influence body size and muscular development. This topic will not be dealt with here.

### 9.6. Tissue-specificity of steroids

**Figure 6.**
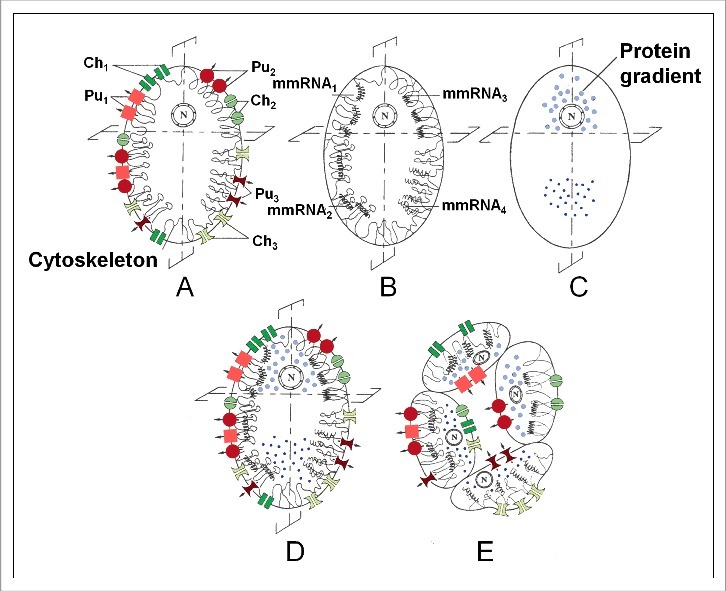
The basic principle underlying differentiation during development that explains in which aspects all cells of a differentiated organism differ from each other. Schematic representation of ‘the double asymmetry principle’ that is instrumental to the essence of development, namely: Keep during the successive mitotic divisions the genome constant, but change over and over again its inorganic ion and/or macromolecular development. Mechanisms instrumental in the functional asymmetry in the stem cell (zygote) of a hypothetical 4-celled, epithelially organized organism (animal) (A-E) (modified after [4,18]. To illustrate the principle, a few membrane proteins, namely ion pumps and channels are depicted. Many other proteins are also present in membranes. First, an asymmetrical distribution is realized (i) of the plasma membrane-cytoskeletal complex (A: Pu1-Pu3 = 3 types of ion pumps; Ch1-Ch3 = 3 types of ion channels); (ii) and/or of maternal messenger RNAs (B: mmRNA1-mmRNA4, some of which can be anchored to the cytoskeleton; (iii) and/or of gradients of certain proteins in the cytoplasm/yolk (C) (e.g. of the bicoid and nanos gradients in eggs of *Drosophila*). Next the egg is cleaved in an asymmetrical way. Sometimes this happens already during the first cleavage, but never later than during the third. In D, which represents the superposition of A, B, and C, it is the second cleavage which will give rise to four different cell types, all sharing an identical genome but differing in their membrane properties (E). Here, only a few proteins residing in the plasma membrane are depicted. In addition many other proteins reside in the intracellular membrane system.

Although sex steroids affect the whole body, they do not generate exactly the same effects in all cell types, For understanding the differential effect of steroids in males versus females, or/and in different tissues, one has to keep in mind the very basic principle of differentiation during embryonic development. It says that all cells of a differentiated organism have the same genome (some epigenetic changes not taken into account), but *differ in their membrane-cytoskeletal properties* (Fig. 4). The double asymmetry principle [18], which is at work during the first cell divisions (cleavage) after fertilization of the egg is a key causal mechanism in this respect (Fig. 6). Although there are only a few different types of sex steroids, in combination with their effects on the highly variable Ca^2+^-homeostasis system, they can help to generate a lot of different behaviours. This is important to keep in mind when we will discuss how many gender types exist.

**Figure 7.**
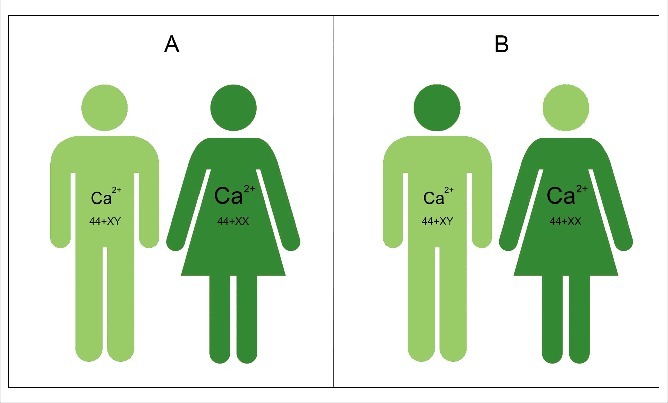
Cartoon illustrating the idea that the main difference between the various gender forms resides in the C^a2+^-homeostasis system, in particular that present in some brain areas. Given that the human brain contains about 100 billion nerve cells, it is *de facto* impossible that two individuals have exactly the same Ca^2+^-homeostasis system in the totality of their brain, even if these two individuals are identical twins. This figure illustrates the commonly observed situation that the sexual thinking and behaviour of transgenders reflects more the situation of the other heterosexual somatic sex than their own somatic genetic sex. Between these two depicted extremes, numerous intermediate forms are theoretically possible. Indeed, it is more likely that not the whole brain but specific brain regions can display (subtle) changes in Ca^2+^ homeostasis with effects on behaviour as a result.

## 10. How many gender forms are theoretically and physiologically possible?

To understand my answer which is: As many as there are sexually reproducing individuals, one has to keep in mind the most fundamental principle of embryonic development that was explained before (Fig. 6).

### 10.1. Hetero-, homo- bisexuality-, …: Major and minor variants of gender

The well-known gender forms are: heterosexual males and females, homosexual males and females, monogamous- and monandric individuals, asexual individuals not interested in sexual activities with other individuals, bisexuals, and a few more. The frequency in the population of individuals who engage in sexual activities with non-humans, with humans who are not in the normal reproductive age category, and transsexuals is very low. Hermaphroditism, the situation in which one individual has both testes and ovaries is normal in some animal species, e.g. in some snails. Such animals behave one day as male and the next day as female: they do not fertilize themselves. Thus, if one adds up these forms, the outcome is that in humans there are about 15 readily observable gender forms. This number drastically increases in species in which mating is not random, but in which individuals only engage in reproductive activities with a chosen partner. In such conditions, thus when gender behaviour becomes very *personalized*, the number of possible gender forms will near the number of all constituting individuals of the whole population. Thus in our own species there are as many different gender variants as there are individuals, 7 billion plus. But the total number of genes coding for proteins present in the human genome is only about 20,000. In addition, there are also some 10,000 pseudogenes, some genes can be subject to epigenetic modifications, and the junk DNA (that does not code for proteins) also has some regulatory functions. Even if all this is taken into account, the discrepancy 7 billion+ versus some 30,000+ genes and pseudogenes, illustrates that gender must have a physiological basis that enables a huge variability that can unfold while making use of only a limited number of genes.

### 10.2. Homosexuality, Trans genders

Quite a lot of research has been devoted to the physiological basis of homosexuality in humans. Because the percentage of homosexuals seems to be rather similar in all cultures (a few percent), and because homosexuality remains constant over generations, a genetic basis has been hypothesized. However, the search for a homosexuality gene did not yield any positive result. Hence it may be concluded that such gene does not exist, as could be expected from the principles of genetics. Indeed, the frequency of such a gene would continuously decrease in a population for lack of progeny. However, that does not rule out that there is a genetic basis, but that the genetics apply to one of the control systems of behaviour that is subject to variability. In my opinion, the complex Ca^2+^- homeostasis system may play a differentiating role in homo-versus heterosexuality, like it does in regular sex differentiation. When an embryo differentiates, all its somatic cells acquire an individualized specific membrane-cytoskeletal system. No two individuals have the same set of brain cells (100 billion nerve cells in fully differentiated human individuals with an incredibly complex network of connections). Ball et al [52]. suggested that that it might be useful to view the brain as a secondary sexual characteristic. Furthermore, it is logical to assume that different brain parts may acquire different sets of Ca^2+^-homeostasis patterns (Fig. 7). *In my opinion, this represents the very physiological basis of gender variability*.

The Dutch Professor Dick Swaab and his team have devoted great efforts in their search for an anatomical and/or physiological basis accounting for differences in the brains of hetero-versus homosexuals. In 2011 Bao and Swaab [53] summarized the state of affairs as follows: During the intrauterine period a testosterone surge masculinizes the foetal brain, whereas the absence of such a surge results in a feminine brain. As sexual differentiation of the brain takes place at a much later stage in development than sexual differentiation of the genitals, these two processes can be influenced independently of each other. Sex differences in cognition, gender identity (heterosexuality, homosexuality or bisexuality), and the risk of developing neuropsychiatric disorders are programmed in our brain during early development. *There is no evidence that one's postnatal social environment plays a crucial role in gender identity or sexual orientation* (end citation). Balthazart [34] also emphasizes the importance of the pre- and perinatal developmental period for establishing sex differences in partner preferences. Thus, if this is the case, one's gender is already fixed before/around birth: it is inborn and not the result of a free-will choice of the individual. No doubt that androgens and estrogens play a key role in gender. However, how they do so is not fully clear, despite decades of research in this domain. My view is that sex steroids influence gender mainly through their effects on Ca^2+^-homeostasis in specific brain areas.

### 10.3. Is one gender form superior over others? Biology says: Physiologically not at all

If one forgets (what one should certainly not do) about the basic principle that there is no goal in evolution, the answer could be that the heterosexual gender variants are superior because they *aim* at producing a progeny. Because, many people believe that reproduction has been created or has come into existence with the *goal of a progeny,* gender variants that do not aim at a progeny, will be perceived as unnatural or, in a religious context, even as sinners. But reproductive archaeology teaches us that sexual reproduction did come into existence as the *result of a sort of bacterial infection*, not at all with the goal of producing a progeny in a novel way, namely by making use of special sex cells (sperm and eggs). Of course, heterosexuality is also in such a context a seemingly more successful form because it enables a progeny. However, this is a fortuitous coincidental feature, which does not imply any (in the case of humans) moral superiority: all gender forms have a cell-physiological basis. They are all variants of the same basic model. The supposedly better (in the long-term) form for the entire population, the heterosexual one, only *seems* better because it occurs in a much larger percentage of the population, and because most people erroneously think that the production of gametes and fertilization has come into existence in the course of evolution with the *goal* of producing a progeny. Evidently without a progeny the population dies.

## 11. Discussion

There is a world, not to say a Universe, of difference in the approach of the concept Gender by biologists versus by sociologists. For biologists, *Homo sapiens* is a placental mammal that shares many characteristics- physiological and behavioural – with other mammalian species. These have been inherited from a common ancestor. Although this is an important issue when one tries to interpret reproduction-related behaviour, this aspect of gender is undervalued in the sociological approach of gender. *Homo* has a complex social behaviour. Occasionally that yields conflicts in society with respect to the allocation of some types of tasks/labour and to hierarchical status. With some exaggeration, biologists active in the exact sciences search for the *Nature* basis underlying gender, while the humanities and in particular sociologists are more interested in the *more fluid Nurture aspects* of gender. This is apparent from the interpretation, often in a feminist context, of reproduction-related behaviour and its economic and political consequences. This duality is reflected in Simone de Beauvoir's phrase [54]: *One is not born, one becomes a woman* with which many biologists will largely disagree. To date, it is generally accepted that it is not *or* but *and*, Nature ( = genetically determined) *and* Nurture (influenced by the environment in the broad sense). Yet, it remains difficult to formulate an appropriate vocabulary and conceptual approach that makes a seamless integration of the two approaches possible.

From the study of comparative physiology of reproduction in vertebrates and invertebrates, it has become clear that many aspects of reproduction involving (hetero) gametes came into existence long ago in evolution, and that they have been very well conserved since [7]. For biologists this means that *gender as a biological concept* not only has a meaning for our own species *Homo sapiens* with its complex social organization, but for numerous other animal species as well. It also means that there must exist an evolutionarily well conserved genetics-based system that codes for key aspects of reproductive behaviour. The link between genetics and sex hormones is documented since long. In this paper, I emphasized the importance for gender in general, and for reproduction-linked behaviour in particular of the Ca^2+^-homeostasis system which is also largely genetics-based. This system is very versatile and dynamic. Due to the frequent occurrence of Ca^2+^-puffs (which are usually very short-lived) in many cell types and in particular in muscle cells, it is not easy to determine the exact [Ca^2+^]i in such cells. Hence my statement that all cells of a differentiated organism differ from each other, is not based upon exact measurements. It is deduced from a theoretical statistics-based argument, namely far too many causal agents play a role.

I can imagine that without a knowledgeable insight into the fundamentals of animal physiology and development, the hypothesis that sexual reproduction results from an evolutionarily ancient sort of prokaryotic (bacterial) infection of a proto-eukaryotic cell that eventually became the common ancestor of all eukaryotes, is at first encounter perceived as both counterintuitive and far-sought. Yet there are sound scientific arguments to support this view [7]. Not only is the reproductive state a disease state, it is not even a mild one, but a death-causing state: *Sex kills* is one of the numerous theories explaining aging phenomena [55]. In addition, the view that the female body, the brain inclusive, is somewhat more *poisoned* by Ca^2+^ than the male one [2] may sound discriminatory towards females in general, but it is not, to the contrary. The explanation is that the beneficial effects Ca^2+^ undoubtedly has in the body result from the fact that short-lived rising Ca^2+^ concentrations ( = toxic puffs) are causal to them, e.g. in biochemical signalling (Ca^2+^ as secondary messenger). As an example, in many species, females live longer than males, despite, or better thanks to the toxicity of Ca^2+^. Females have better means to remove excess Ca^2+^ from their body, and can therefore better cope with the toxicity of Ca^2+^. Yet, decalcification of the skeleton resulting in osteoporosis that affects woman more than man is a negative aspect.

The interplay between genetics, sex hormones, Ca^2+^-homeostasis, the social environment and other aspects of Nurture enables a huge variability in gender variants. In this context, a major gender-related topic of discussion is the place of homosexuality in an animal population, and the way this is perceived in human society in particular. It is known since long that homosexuality, one of the many possible gender variants, occurs in several animal species. For biologists this illustrates that this gender form must have a physiological basis that occurs widely in the animal kingdom, and that can generate variability in reproduction-related behaviour. The key actors in Ca^2+^-homeostasis are proteins that reside in membranes: Ca^2+^ channels and Ca^2+^-pumps. Bao and Swaab (2013) attribute little weight to the role of post-partum Nurture in steering the coming into existence of gender orientation, e.g. hetero- versus homosexuality. I support this view: Nature, thus genetics, is much more important than Nurture in defining gender and gender orientation. Differential sex steroid production and differential Ca^2+^-homeostasis are already effective long before birth.

In conclusion: In this paper I advanced the physiology-based view that *there are probably as many different gender variants as there are sexually reproducing individuals,* and this not only in humans. Hence, there is no need to make subcategories in the gender variants with the purpose to install a moral hierarchy as they are all *physiologically* equal. This does not mean that all gender variants contribute equally well to the well-being of the whole population. I hope that growing awareness that the Ca^2+^- homeostasis system which is a key signalling system in all eukaryotes, acts as one of the corner stones on which gender is based, may contribute to a better acceptance of the variability of in gender forms as a natural, largely genetic (inborn) system.
